# New Innovations in Wound Healing and Repair

**DOI:** 10.3390/ijms20071724

**Published:** 2019-04-08

**Authors:** Allison J. Cowin

**Affiliations:** Regenerative Medicine, Future Industries Institute, University of South Australia, Adelaide SA 5095, Australia; Allison.Cowin@unisa.edu.au; Tel.: +61-8302-5018

Wounds are a largely unrecognized, spiraling epidemic that affect millions of people world-wide. They are complex and involve the temporal and spatial involvement of many different cell types and tissue processes. Recent advances in our understanding of wound repair and regeneration, as well as the many novel and exciting approaches aimed at healing chronic/acute wounds and reducing scar formation, have made this a pertinent time for a Special Issue aimed at overviewing this important field.

The goal of this Special Issue was to provide a summary of the field to describe its impact, as well as to introduce the recent advances in understanding the mechanisms that underpin wound healing and scar formation. This Special Issue achieved this goal with the inclusion of 19 outstanding papers that highlight the innovative approaches researchers from around the world are undertaking to improve healing and reduce scar formation. It includes articles that outline new approaches to reduce wound infection [1,2] as well as studies aimed at developing new in vitro chronic wound models as alternatives to animal experimentation [3]. The interest in natural products as potential wound agents is evidenced through three articles that investigated healing responses to honey [4], deoxyshikonin [5], and anthocyanins extacted from *Oryza sativa L* [6]. Understanding cytoskeletal interactions in keratinocytes and fibroblasts [7] as well as neutrophil proteome signatures [8] also provide important new information that may inform researchers with the development of new therapeutic approaches to improve healing responses. Altering TLR [9] and inflammasome signaling [10] provide further important insights into healing responses. As inflammation is a critical part of the wound healing process these papers help us to understand and therefore open up opportunities to manipulate the inflammatory response that will pave the way for improved healing in the future. By disseminating and sharing our research, we ensure that future advances in the field can be made. These articles provide a wealth of new knowledge that may spark the next new innovation that will make a difference for people who suffer from impaired healing.

## References

[1] Kawahara T., Takita M., Masunaga A., Morita H., Tsukatani T., Nakazawa K., Go D., Akita S. (2019). Fatty Acid Potassium Had Beneficial Bactericidal Effects and Removed *Staphylococcus aureus* Biofilms while Exhibiting Reduced Cytotoxicity towards Mouse Fibroblasts and Human Keratinocytes. Int. J. Mol. Sci..

[2] Johnson T.R., Gómez B.I., McIntyre M.K., Dubick M.A., Christy R.J., Nicholson S.E., Burmeister D.M. (2018). The Cutaneous Microbiome and Wounds: New Molecular Targets to Promote Wound Healing. Int. J. Mol. Sci..

[3] Caley M., Wall I.B., Peake M., Kipling D., Giles P., Thomas D.W., Stephens P. (2018). Development and Characterisation of a Human Chronic Skin Wound Cell Line—Towards an Alternative for Animal Experimentation. Int. J. Mol. Sci..

[4] Martinotti S., Laforenza U., Patrone M., Moccia F., Ranzato E. (2019). Honey-Mediated Wound Healing: H_2_O_2_ Entry through AQP3 Determines Extracellular Ca^2+^ Influx. Int. J. Mol. Sci..

[5] Park J.Y., Shin M.-S., Hwang G.S., Yamabe N., Yoo J.-E., Kang K.S., Kim J.-C., Lee J.G., Ham J., Lee H.L. (2018). Beneficial Effects of Deoxyshikonin on Delayed Wound Healing in Diabetic Mice. Int. J. Mol. Sci..

[6] Tancharoen S., Shakya P., Narkpinit S., Dararat P., Kikuchi K. (2018). Anthocyanins Extracted from *Oryza sativa* L. Prevent Fluorouracil-Induced Nuclear Factor-κB Activation in Oral Mucositis: In Vitro and In Vivo Studies. Int. J. Mol. Sci..

[7] Kopecki Z., Stevens N.E., Yang G.N., Melville E., Cowin A.J. (2018). Recombinant Leucine-Rich Repeat Flightless-Interacting Protein-1 Improves Healing of Acute Wounds through Its Effects on Proliferation Inflammation and Collagen Deposition. Int. J. Mol. Sci..

[8] Bekeschus S., Lackmann J.-W., Gümbel D., Napp M., Schmidt A., Wende K. (2018). A Neutrophil Proteomic Signature in Surgical Trauma Wounds. Int. J. Mol. Sci..

[9] Lewandowska-Polak A., Brauncajs M., Jarzębska M., Pawełczyk M., Kurowski M., Chałubiński M., Makowska J., Kowalski M.L. (2018). Toll-Like Receptor Agonists Modulate Wound Regeneration in Airway Epithelial Cells. Int. J. Mol. Sci..

[10] Lee J.S., Robertson A.A.B., Cooper M.A., Khosrotehrani K. (2018). The Small Molecule NLRP3 Inflammasome Inhibitor MCC950 Does Not Alter Wound Healing in Obese Mice. Int. J. Mol. Sci..

